# Root Abscisic Acid Contributes to Defending Photoinibition in Jerusalem Artichoke (*Helianthus tuberosus* L.) under Salt Stress

**DOI:** 10.3390/ijms19123934

**Published:** 2018-12-07

**Authors:** Kun Yan, Tiantian Bian, Wenjun He, Guangxuan Han, Mengxue Lv, Mingzhu Guo, Ming Lu

**Affiliations:** 1 Key Laboratory of Coastal Environmental Processes and Ecological Remediation, Yantai Institute of Coastal Zone Research, Chinese Academy of Sciences, Yantai 264003, China; czlbtt@163.com (T.B.); wjhe@yic.ac.cn (W.H.); xuhualing1981@163.com (M.L.); 2 School of Life Sciences, Ludong University, Yantai 264025, China; 3 College of Life Sciences, Yantai University, Yantai 264005, China; m17616156626@163.com (M.G.); 17865561399@163.com (M.L.)

**Keywords:** chlorophyll fluorescence, lipid peroxidation, Na^+^, photosynthesis, photosystem

## Abstract

The aim of the study was to examine the role of root abscisic acid (ABA) in protecting photosystems and photosynthesis in Jerusalem artichoke against salt stress. Potted plants were pretreated by a specific ABA synthesis inhibitor sodium tungstate and then subjected to salt stress (150 mM NaCl). Tungstate did not directly affect root ABA content and photosynthetic parameters, whereas it inhibited root ABA accumulation and induced a greater decrease in photosynthetic rate under salt stress. The maximal photochemical efficiency of PSII (Fv/Fm) significantly declined in tungstate-pretreated plants under salt stress, suggesting photosystem II (PSII) photoinhibition appeared. PSII photoinhibition did not prevent PSI photoinhibition by restricting electron donation, as the maximal photochemical efficiency of PSI (ΔMR/MR_0_) was lowered. In line with photoinhibition, elevated H_2_O_2_ concentration and lipid peroxidation corroborated salt-induced oxidative stress in tungstate-pretreated plants. Less decrease in ΔMR/MR_0_ and Fv/Fm indicated that PSII and PSI in non-pretreated plants could maintain better performance than tungstate-pretreated plants under salt stress. Consistently, greater reduction in PSII and PSI reaction center protein abundance confirmed the elevated vulnerability of photosystems to salt stress in tungstate-pretreated plants. Overall, the root ABA signal participated in defending the photosystem’s photoinhibition and protecting photosynthesis in Jerusalem artichoke under salt stress.

## 1. Introduction

Soil salinity is a serious problem for agricultural cultivation because of the detrimental effects on crop growth and yield. Under salt stress, plants have to tolerate osmotic stress, ionic toxicity, and secondary oxidative stress, and the metabolisms may be disrupted with damaged biological macromolecules [1,2,3]. Correspondently, plants have evolved some physiological adaption measures such as Na^+^ exclusion, osmolyte synthesis, and antioxidant induction, however, signal molecules which sensitively perceive external stresses are required to activate these protective mechanisms [4,5].

Abscisic acid (ABA) is defined as a stress hormone, because ABA can mediate extrinsic stress signals to improve expression of resistance genes [6,7,8]. As well documented, ABA plays an important role in regulating stomatal closure to limit water loss from transpiration, which assists in plant acclimatization to osmotic tolerance [8,9,10]. The positive role of ABA in plant salt tolerance also has been reviewed, and besides stomatal closure, osmolyte synthesis and antioxidant induction usually associate with ABA signal under salt stress despite some inconsistent reports due to species difference [7,11,12,13,14]. Na^+^ is the primary toxic component for plants upon salt stress [2]. To date, it is still ambiguous whether ABA signal contributes to controlling Na^+^ long-distance transportation and exclusion [4,15]. Particularly, Cabot et al. [16] reported that leaf ABA accumulation resulted in higher leaf Na^+^ concentration in *Phaseolus vulgaris* under salt stress due to lowered leaf Na^+^ exclusion and increased Na^+^ translocation from root to shoot. Therefore, ABA function in defending salt-induced ionic toxicity seems not definite in contrast to its role in osmotic tolerance. Moreover, it remains unknown whether root ABA or leaf ABA has a greater effect on plant salt tolerance.

As one of the most important metabolisms for plant growth, photosynthesis is sensitive to salt stress. Photosynthetic analysis seems to be an effective and convenient way for diagnosing plant salt tolerance, because photosynthetic capacity in susceptible cultivars is more liable to be inhibited than tolerant ones [17,18,19,20,21,22]. Salt-induced stomatal closure initially depressed photosynthesis by lowering CO_2_ availability [23,24], and subsequently, the negative effect on Rubisco can further restrict CO_2_ fixation [25,26]. Eventually, the declined CO_2_ assimilation will elevate excitation pressure in chloroplast through feedback inhibition on photosynthetic electron transport and then bring about photosystems photoinhibition or even irreversible damage with excess ROS production [27,28]. At present, photosystems photoinhibition and interaction under salt stress have been reported. In addition to PSII, PSI is also a crucial photoinhibition site and PSI photoinhibition poses a great threat to the entire photosynthetic apparatus by inducing PSII photoinhibition [20,29]. However, the relationship between the ABA signal and photosystem photoinhibition remains to be disclosed. ABA-induced stomatal limitation may trigger photosystem photoinhibition, but ABA-induced antioxidant activity can prevent from photoinhibition by scavenging reactive oxygen species (ROS). Particularly, the ambiguous function of ABA for regulating Na^+^ transportation make it more complex.

Jerusalem artichoke (*Helianthus tuberosus* L.) is a valuable energy crop with high fructose and inulin concentrations in the tuber [30]. Jerusalem artichoke has certain salt tolerance and serves as a promising crop for utilizing coastal marginal land in China [30,31]. According to previous studies, salt stress could induce photosynthetic stomatal limitation, oxidative injury, chlorophyll loss, and ABA accumulation in Jerusalem artichoke [32,33,34]. However, the importance of ABA for salt tolerance in Jerusalem artichoke has not been tested. At present, gas exchange combined with modulated chlorophyll fluorescence has become a traditional method to examine plant stress tolerance. Recently, a simultaneous measurement of chlorophyll fluorescence transients and modulated 820 nm reflection has been applied to investigate PSII and PSI performance and their coordination, which enriches the traditional photosynthetic analysis [20,35,36,37,38]. In this study, we aimed to verify ABA function for salt adaptability in Jerusalem artichoke by photosynthetic analysis after applying a specific ABA synthesis inhibitor to the roots. Simultaneous measurement of chlorophyll fluorescence transients and modulated 820 nm reflection was carried out to complement traditional gas exchange analysis for revealing photosystems performance and coordination. Particularly, the abundance of PSII and PSI reaction center proteins was detected by immunoblot analysis to confirm salt-induced damage on photosystems. We hypothesized that root ABA accumulation helped prevent photosystems photoinhibition and protect photosynthesis by alleviating water loss and ionic toxicity. Our study can deepen the knowledge of salt tolerance in Jerusalem artichoke and may provide a reference for the cultivation in coastal saline land.

## 2. Results

### 2.1. Leaf Na^+^, Relative Water, Malondialdehyde (MDA) and H_2_O_2_ Content, and Root Na^+^ Flux

After four days of salt stress, leaf Na^+^ and H_2_O_2_ content were significantly increased, whereas leaf relative water content was significantly decreased (Table 1). Leaf Na^+^, MDA and H_2_O_2_ content, and root Na^+^ flux were not directly affected by tungstate. Upon four days of salt stress, tungstate had no effect on the decreased amplitude of leaf relative water content but amplified the increase in leaf Na^+^ and H_2_O_2_ content (Table 1). Salt-induced significant increase in leaf MDA content was found in tungstate-pretreated plants rather than non-pretreated plants (Table 1). Root Na^+^ efflux was significantly elevated by salt stress, but salt-induced increase in Na^+^ efflux was greatly reduced in tungstate-pretreated plants (Table 1).

### 2.2. ABA Content in Leaf and Root

**Figure 1:**
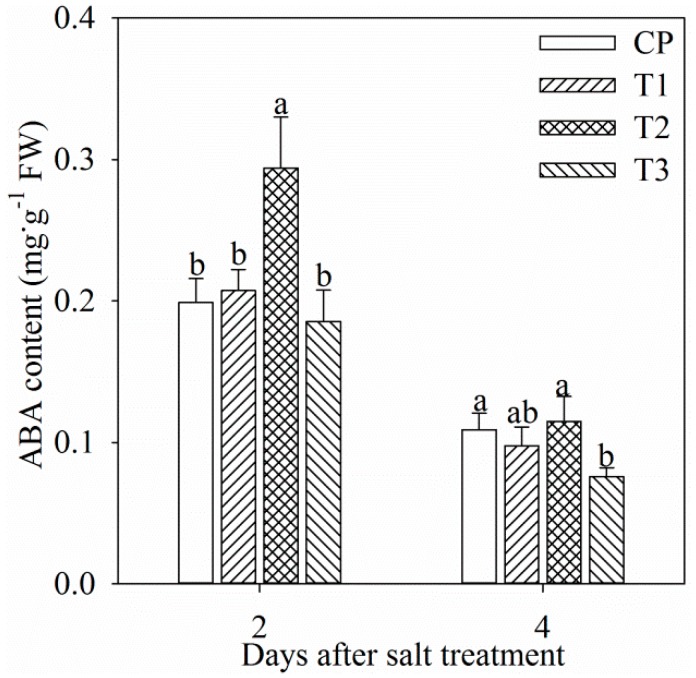
Changes in root abscisic acid (ABA) content in Jerusalem artichoke after salt stress for two days (a) and four days (b). Data in the figure indicate mean of five replicates (±SD), and different letters on error bars indicate significant difference at *p* < 0.05. CP indicates control plants without pretreatment and NaCl stress; T1 indicates tungstate-pretreated plants without NaCl stress; T2 indicates non-pretreated plants under 150 mM NaCl stress; T3 indicates tungstate-pretreated plants under 150 mM NaCl stress. The symbols, CP, T1, T2, and T3 are also used in the following figures.

Single tungstate pretreatment did not affect root ABA content (Figure 1). After two days of salt stress, root ABA content was significantly increased by 47.8%, and the increase was dampened by tungstate pretreatment (Figure 1). After four days of salt stress, root ABA content was still remarkably lower in tungstate-pretreated plants than non-pretreated plants under salt stress (Figure 1). In all treatment groups, root ABA content after four days was lower than that after two days (Figure 1), which might originate from root development or ABA translocation from root to leaf.

### 2.3. Gas Exchange and Modulated Chlorophyll Fluorescence Parameters

**Figure 2:**
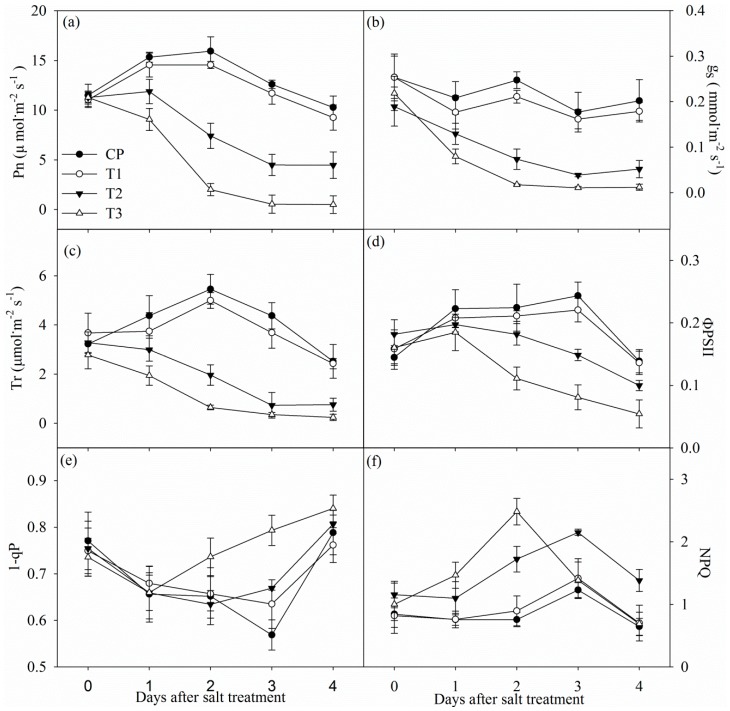
Changes in photosynthetic rate (Pn, (**a**)), stomatal conductance (g_s_, (**b**)), transpiration (Tr, (**c**)), actual photochemical efficiency of PSII (ΦPSII, (**d**)), PSII excitation pressure (1-qP, (**e**)) and non-photochemical quenching (NPQ, (**f**)) in Jerusalem artichoke under salt stress. Data in the figure indicate the mean of five replicates (±SD).

Tungstate did not obviously influenced photosynthetic rate (Pn), stomatal conductance (g_s_) and transpiration rate (Tr) (Figure 2a–c). Pn, g_s_, and Tr significantly decreased in non-pretreated plants after one day of salt stress, and the decrease was up to 56.54%, 74.31% and 76.86% after four days of salt stress. In contrast, the decrease in Pn, g_s_ and Tr was remarkably higher in tungstate-pretreated plants upon salt stress (Figure 2a–c).

Under salt stress, decreased actual photochemical efficiency of PSII (ΦPSII) was noted with increased non-photochemical quenching (NPQ) in non-pretreated plants, whereas PSII excitation pressure (1-qP) did not show obvious change (Figure 2d–f). Tungstate did not significantly influenced ΦPSII, 1-qP and NPQ, and salt-induced decrease in ΦPSII was greater in tungstate-pretreated plants than non-pretreated plants. After two and three days of salt stress, 1-qP was significantly increased in tungstate-pretreated plants, but the increase became slight after four days of salt stress (Figure 2e). NPQ was significantly increased in tungstate-pretreated plants after one day of salt stress, but the increase disappeared after 3 days of salt stress (Figure 2f).

### 2.4. Chlorophyll Fluorescence and Modulated 820 nm Reflection Transients

**Figure 3:**
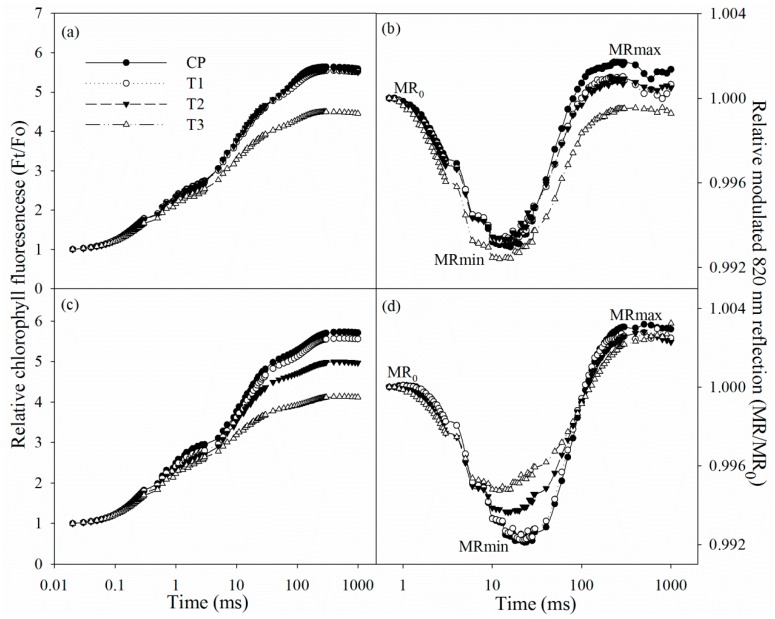
Chlorophyll fluorescence transients and 820 reflection transients during the first 1 s red illumination in Jerusalem artichoke under salt stress for two days (**a**,**b**) and four days (**c**,**d**). Ft is chlorophyll fluorescence intensity during the 1 s of red illumination, and Fo is fluorescence intensity at 20 µs, when all reaction centers of PSII are open. MR is the reflection signal during the 1 s of red illumination, and MR_0_ is the value of modulated 820 nm reflection at the onset of red light illumination (0.7 ms, the first reliable MR measurement). MRmin and MRmax indicate the maximal point during PSI oxidation and the maximal point during PSI re-reduction, respectively. Data in the figure indicate the mean of five replicates.

After two days of salt stress, chlorophyll fluorescence and modulated 820 nm reflection transients did not exhibit obvious change. The initial decrease in 820 nm reflection signal indicated PSI oxidation process, and the subsequent increase suggested that PSI was gradually re-reduced. After two days of salt stress, chlorophyll fluorescence transient descended in tungstate-pretreated plants (Figure 3a), suggesting PSII capacity was negatively affected. After salt stress for two days, the 820 nm reflection transient also changed in tungstate-pretreated plants, indicated by prolonged PSI oxidation process and lowered PSI re-reduction level (Figure 3b).

After four days of salt stress, chlorophyll fluorescence transient declined, while the PSI oxidation process was shortened (Figure 3c,d). Tungstate pretreatment never induced any change in chlorophyll fluorescence and 820 nm reflection transients, but their variations under salt stress were amplified by tungstate pretreatment (Figure 3c,d).

### 2.5. PSII Performance, the Maximal Photochemical Capacity of PSI, and Immunoblot Analysis

**Figure 4:**
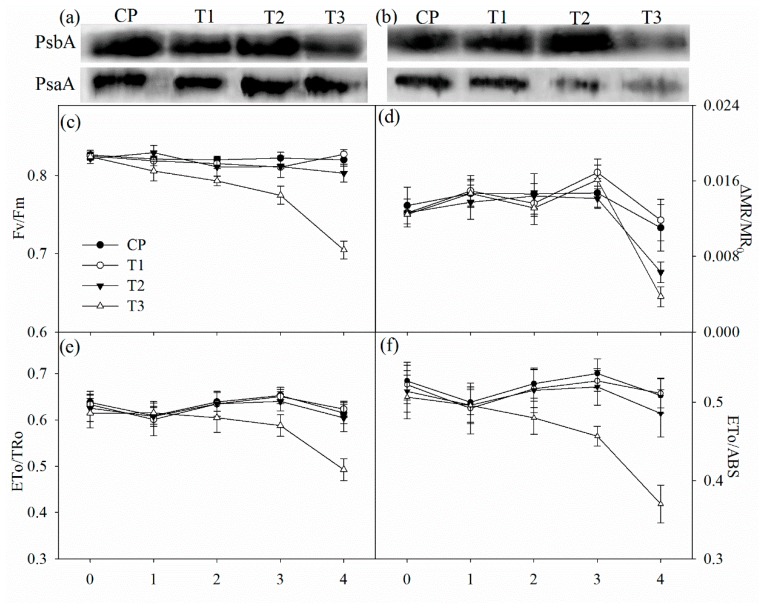
Immunoblot analysis of PSII reaction center protein (PsbA) and PSI reaction center protein (PsaA) abundance after two days (**a**) and four days (**b**) of salt stress and salt-induced changes in the maximal photochemical efficiency of PSII (Fv/Fm, (**c**)) and PSI (ΔMR/MR_0_, (**d**)), probability that an electron moves further than Q_A_ (ETo/TRo, (**e**)) and quantum yield for electron transport (REo/ETo, (**f**)) in Jerusalem artichoke. Data in the figure indicate the mean of five replicates (±SD).

Tungstate had no direct effect on the maximal photochemical capacity of PSI (ΔMR/MR_0_), the maximal quantum yield of PSII (Fv/Fm), probability that an electron moves further than primary acceptor of PSII (ETo/TRo) and quantum yield for electron transport (ETo/ABS) (Figure 4c–f). After two days of salt stress, Fv/Fm, ΔMR/MR_0_, ETo/TRo and ETo/ABS did not obviously change in non-pretreated plants, but significant decrease in Fv/Fm was observed in tungstate-pretreated plants (Figure 4c–f). After four days of salt stress, significant decrease in ΔMR/MR_0_ appeared with slightly lowered Fv/Fm, ETo/TRo and ETo/ABS in non-pretreated plants, but the decrease was greater in tungstate-pretreated plants (Figure 4c–f).

The amount of PSII reaction center protein (PsbA) was reduced in tungstate-pretreated plants after two days of salt stress, and the reduction became more obvious after four days of salt stress (Figure 4a,b). In contrast, PsbA abundance was not affected by salt stress in plants without tungstate pretreatment (Figure 4a,b). After four days of salt stress, PSI reaction center protein (PsaA) abundance was decreased, and the decrease was greater in tungstate-pretreated than non-pretreated plants (Figure 4b).

## 3. Discussion

As with common knowledge, salt stress elevated root ABA concentration in Jerusalem artichoke, and tungstate pretreatment prevented salt-induced root ABA accumulation (Figure 1). The salt-induced greater decrease in Pn and ΦPSII in tungstate-pretreated plants than non-pretreated plants suggested that root ABA aided in protecting photosynthetic process in Jerusalem artichoke against salt stress (Figure 2a,d). Under salt stress, leaf stomatal closure reduced water loss from transpiration in Jerusalem artichoke (Figure 2b,c), but could inevitably induce stomatal limitation on photosynthesis. Tungstate-pretreated plants should encounter stronger photosynthetic stomatal limitation under salt stress due to the greater decrease in g_s_ compared with non-pretreated plants (Figure 2b). Lowered CO_2_ assimilation can elevate PSII excitation pressure by feedback inhibition on photosynthetic electron transport and cause oxidative injury with excessive ROS production [28,39]. Under salt stress, PSII excitation pressure did not obviously change in spite of lowered CO_2_ assimilation in non-pretreated plants, as the excessive excitation energy was effectively dissipated as heat (Figure 2e,f). In contrast, elevated PSII excitation pressure due to greater lowered CO_2_ assimilation and insufficient heat dissipation could bring about photosystems photoinhibition in tungstate-pretreated plants upon salt stress. Notably, elevated PSII excitation pressure disappeared in tungstate-pretreated plants after four days of salt stress (Figure 2e), implying tremendous decrease in trapped energy in reaction center due to severe PSII photoinhibition.

In line with the above deduction, PSII photoinhibition actually occurred in tungstate-pretreated plants upon salt stress, indicated by declined Fv/Fm and chlorophyll fluorescence transient (Figure 3a,c and Figure 4c). Thus, considering slight change in Fv/Fm and chlorophyll fluorescence transient in non-pretreated plants (Figure 3a,c and Figure 4c), root ABA should participate in protecting PSII against photoinhibition in Jerusalem artichoke under salt stress. This positive role of root ABA was corroborated by immunoblot analysis, as lowered and unchanged PsbA abundance appeared, respectively, in tungstate-pretreated and non-pretreated plants upon salt stress (Figure 4a,b). Similar to PSII, PSI photoinhibition also derives from oxidative injury on reaction center proteins [27,28,36]. Along with elevated ROS production and lipid peroxidation (Table 1), PSI photoinhibition appeared after four days of salt stress, indicated by the significant decrease in ΔMR/MR_0_ (Figure 3d). In agreement with our recent study on waterlogging [40], PSI was also more vulnerable than PSII in Jerusalem artichoke under salt stress according to less decrease in Fv/Fm than ΔMR/MR_0_ (Figure 3c,d). Nonetheless, inhibition on root ABA synthesis led to higher PSII susceptibility to salt stress compared with PSI, as earlier significant decrease was observed in Fv/Fm rather than ΔMR/MR_0_ in tungstate-pretreated plants (Figure 3c,d). Prolonged PSI oxidation and lowered PSI re-reduction level in 820 nm reflection transients after two days of salt stress verified greater PSII vulnerability (Figure 2b). Thus, contrary to recent studies [20,29], PSII photoinhibition was not induced by PSI photoinhibition in tungstate-pretreated plants under salt stress. We inferred that photoprotective mechanisms were not adequately induced by salt stress in tungstate-pretreated plants and, as a result, lower heat dissipation appeared with greater excitation pressure on PSII (Figure 2e,f). As a traditional viewpoint, PSII photoinhibition can protect PSI against photoinhibition by restricting photosynthetic electron transport to PSI. In this study, PSII photoinhibition declined electron flow to PSI in tungstate-pretreated plants under salt stress, but PSI photoinhibition was still exacerbated according to greater decrease in ΔMR/MR_0_ compared with non-pretreated plants (Figure 4c–f). After four days of salt stress, greater shortened PSI oxidation also implied more severe PSI photoinhibition in tungstate-pretreated plants (Figure 3d), and this result was confirmed by salt-induced greater reduction in PsaA abundance in tungstate-pretreated plants (Figure 4b). Overall, root ABA signal helped defend salt-induced PSII and PSI photoinhibition in Jerusalem artichoke, and the protective way for PSI did not depend on PSII inactivation.

Although osmotic pressure can rapidly depress photosynthesis through stomatal limitation, Na^+^ toxicity is more hazardous under salt stress. Na^+^ can irreversibly inactivate PSII and PSI by inducing secondary oxidative injury or through direct damage on photosynthetic proteins [41,42,43,44]. Particularly, severe PSII photoinhibition without elevated excitation pressure in tungstate-pretreated plants after four days of salt stress may result from the direct effect of Na^+^ in large part. In this study, inhibition on root ABA synthesis did not influence leaf water status in Jerusalem artichoke under salt stress, as similar relative leaf water content existed in tungstate-pretreated and non-pretreated plants (Table 1). In contrast, inhibited root ABA accumulation declined Na^+^ exclusion from roots and led to prominent increase in leaf Na^+^ concentration (Table 1). Therefore, Na^+^ toxicity should be responsible for more severe PSII and PSI photoinhibition in tungstate-pretreated plants. However, the signal pathway for regulating Na^+^ transport and uptake needs to be revealed in future study.

In agreement with the hypothesis, root ABA signal contributed to defending photosystems photoinhibition and protecting photosynthesis in Jerusalem artichoke under salt stress, but this positive role of root ABA was actualized mainly by reducing Na^+^ toxicity.

## 4. Materials and Methods

### 4.1. Plant Material and Treatment

Tubers of Jerusalem artichoke were collected in Laizhou Bay, China. The tubers were planted in plastic pots filled with vermiculite (one tuber in each pot) and placed in an artificial climatic room (Qiushi, China). The vermiculite was kept wet by watering. In the room, day/night temperature and humidity were controlled at 25/18 °C and 70%, and photon flux density was 400 μmol·m^−2^ s^−1^ for 12 h per day from 07:00 to 19:00. After one month, the tubers germinated and were daily watered with Hoagland nutrient solution (pH 5.7). One month later, health and uniform plants were selected and separated to four groups. In the first group, plants without tungstate sodium pretreatment were not subjected to NaCl stress. In the second group, plants were pretreated with tungstate sodium but not subjected to NaCl stress. In the third group, plants were exposed to 150 mM NaCl for four days without tungstate sodium pretreatment. In the fourth group, plants were pretreated with tungstate sodium and then subjected to 150 mM NaCl for four days. NaCl was added to nutrient solution incrementally by 50 mM step every day to reach the final concentration. The solution was refreshed every two days, and before refreshing solution, the culture substrate was thoroughly leached using nutrient solution for avoiding ion accumulation. One day before salt treatment, tungstate sodium (1 mM), a specific inhibitor of ABA synthesis, was added to nutrient solution for pretreatment.

### 4.2. Measurements of Na^+^, Relative Water Content, and Root Na^+^ Flux

The extraction of Na^+^ was performed according to Song et al. [45]. Deionized H_2_O (25 mL) was added to 0.1 g dried leaf powder and boiled for 2 h. The supernatant was diluted 50 times with deionized H_2_O for measuring Na^+^ content by using an atomic absorption spectrophotometer (TAS-990, Beijing, China). Net Na^+^ flux was measured using NMT (Younger, Amherst, MA, USA) and the principle and protocol for measuring root Na^+^ flux have been elucidated in detail in our recent study [20]. In this experiment, newly developed root segments were sampled and a vigorous Na^+^ flux was identified at 500 µm from the root apex. The measured root position can be visualized under microscope, and tungstate pretreatment dampened salt-induced increase in root Na^+^ efflux (Supplemental Figure S1). The average value of Na^+^ flux is presented in Table 1.

Fresh leaves were harvested and weighed (fresh weight, FW), and then were immersed in distilled water for 4 h at room temperature to determine saturated fresh weight (SW). Subsequently, the leaves were dried completely in an oven at 70 °C and weighed (dry weight, DW). Relative water content (RWC) was calculated as: RWC = (FW − DW)/(SW − DW) × 100%.

### 4.3. Measurements of MDA, H_2_O_2_, and ABA Content

Leaf tissues (0.5 g) were ground under liquid nitrogen and homogenized in 5 mL 0.1% TCA. The homogenate was centrifuged at 12,000× *g* and 4 °C for 10 min to collect the supernatant for measurements of MDA and H_2_O_2_ content. The supernatant (0.5 mL) was mixed with 10 mM potassium phosphate buffer (0.5 mL, pH 7.0) and 1 M KI (1 mL), and the absorbance at 390 nm was recorded for calculating H_2_O_2_ content [46]. MDA content was determined by thiobarbituric acid reaction to reflect the extent of lipid peroxidation [47].

ABA content was analyzed according to Lopez-Carbonell and Jauregui [48] with some modification. Root and leaf tissues (0.5 g) were ground under liquid nitrogen and homogenized in 3 mL 80% methanol containing 0.1% acetic acid. After agitation for 30 min at 4 °C, the homogenate was centrifuged at 12,000× *g* and 4 °C for 10 min. The supernatant was filtered through a 0.45 µm polytetrafluoroethylene membrane, and the filtrate (10 µL) was injected into a high performance liquid chromatography instrument equipped with mass spectrometer (Thermo, Waltham, MA, USA). A hypersil C18 column (4.6 mm × 150 mm; particle size, 5.0 µm) was used in the liquid chromatography system, and the mobile phase consisted of water with 0.1% HCO_2_H (A) and MeOH with 0.1% HCO_2_H (B). A gradient elution program was applied, and the initial gradient of methanol was kept at 30% for 2 min and increased linearly to 100% at 20 min. All the analyses of mass spectrum (MS) were performed using ionspray source in negative ion mode, and MS/MS product ions were produced by collision-activated dissociation of selected precursor ions. Since many compounds could present the same nominal molecular mass, MS/MS method was required to selectively monitor ABA in crude plant extracts by identifying parent mass and unique fragment ion. In this study, MS/MS method was used for the quantitation of ABA by monitoring 263/153 transition, and ABA concentration was determined by using a standard curve plotted with known concentrations of the standards.

### 4.4. Measurements of Gas Exchange and Modulated Chlorophyll Fluorescence

Gas exchange and modulated chlorophyll fluorescence were simultaneously measured by using an open photosynthetic system (LI-6400XTR, Li-Cor, Lincoln, NE, USA) equipped with a fluorescence leaf chamber (6400-40 LCF, Li-Cor). Temperature, CO_2_ concentration and actinic light intensity were, respectively, set at 25 °C, 400 μmol·mol^−1^ and 1000 μmol·m^−2^ s^−1^ in the leaf cuvette. Pn, g_s_ and Tr were simultaneously noted. After steady-state fluorescence yield was recorded, a saturating actinic light pulse of 8000 μmol·m^−2^ s^−1^ for 0.7 s was used to produce maximum fluorescence yield by temporarily inhibiting PSII photochemistry for measuring ΦPSII. Photochemical quenching coefficient was also recorded for calculating 1-qP. Thereafter, the leaves were dark-adapted for 30 min, and a saturating actinic light pulse of 8000 μmol·m^−2^ s^−1^ for 0.7 s was applied to measure the maximal fluorescence for calculating NPQ [49].

### 4.5. Simultaneous Measurements of Chlorophyll Fluorescence and Modulated 820 nm Reflection Transients

A multifunctional plant efficiency analyzer (MPEA, Hansatech, Norfolk, UK) was used for the measurements, and its operating mechanism has been described in detail [37]. The leaves were dark-adapted for 30 min, and the leaves were orderly illuminated with 1 s red light (627 nm, 5000 μmol photons·m^−2^ s^−1^), 10 s far red light (735 nm, 200 μmol photons·m^−2^ s^−1^) and 2 s red light (627 nm, 5000 μmol photons·m^−2^ s^−1^). Chlorophyll fluorescence and modulated 820 nm reflection were simultaneously detected during the illumination. Chlorophyll fluorescence and modulated 820 nm reflection transients were simultaneously recorded during the illumination. Fv/Fm, ETo/TRo, and ETo/ABS were calculated according to chlorophyll fluorescence transients [20], and ΔMR/MR_0_ was determined from modulated 820 nm reflection signal [50,51,52].

### 4.6. Isolation of Thylakoid Membranes and Western Blot

Five grams of leaf discs were ground under liquid nitrogen and homogenized in a solution containing 400 mM sucrose, 50 mM HEPES-KOH (pH 7.8), 10 mM NaCl, and 2 mM MgCl_2_ [53]. The homogenate was filtered through two layers of cheesecloth and then centrifuged at 5000× *g* and 4 °C for 10 min to collectthylakoid pellets. The pellets were resuspended in the homogenization buffer, and chlorophyll content was measured.

Thylakoid membranes with 10 μg chlorophyll were separated by a 12% (*w*/*w*) SDS-PAGE gel. Proteins from the gel were transferred onto polyvinylidene fluoride membrane by semi dry method. After blocking with 5% skimmed milk for 1 h, the membranes were incubated for 2 h with the primary anti-PsbA or anti-PsaA antibodies (PhytoAB, San Francisco, CA, USA) and then incubated with horseradish peroxidase-conjugated anti-rabbit IgG antibody (PhytoAB, USA) for 2 h. BeyoECL Plus substrate (Beyotime Biotechnology, Shanghai, China) was used to test immunoreaction, and the chemiluminescence was detected by a Tanon-5500 cooled CCD camera (Tanon, Shanghai, China).

### 4.7. Statistical Analysis

One-way ANOVA was carried out by using SPSS 16.0 (SPSS Inc., Chicago, IL, USA) for all sets of data. The values presented are the means of measurements with five replicate plants, and comparisons of means were determined through LSD test. The difference was considered significant at *p* < 0.05.

## Figures and Tables

**Table 1:** H_2_O_2_, malondialdehyde (MDA), Na^+^ and relative water contents in the leaf and average root Na^+^ flux in Jerusalem artichoke after four days of salt stress. Data in the table indicate the mean of five replicates (±SD). Within each column, means followed by the same letters are not significantly different at *p* < 0.05. FW indicates fresh weight. CP indicates control plants without pretreatment and NaCl stress; T1 indicates tungstate-pretreated plants without NaCl stress; T2 indicates non-pretreated plants under 150 mM NaCl stress; T3 indicates tungstate-pretreated plants under 150 mM NaCl stress.

Treatments	H_2_O_2_ Content (μmol·g^−1^ FW)	MDA Content (nmol·g^−1^ FW)	Na^+^ Content (mg·g^−1^ FW)	Root Na^+^ Efflux (pmol·cm^−2^ s^−1^)	Relative Water Content (%)
CP	0.11 ± 0.01c	53.00 ± 5.86b	1.08 ± 0.20c	1.80 ± 0.70c	91.41 ± 3.55a
T1	0.10 ± 0.02c	54.13 ± 5.64b	1.23 ± 0.44c	3.84 ± 1.09c	90.30 ± 1.92a
T2	0.18 ± 0.04b	52.83 ± 4.11b	3.58 ± 0.25b	130.13 ± 23.59a	62.93 ± 5.78b
T3	0.27 ± 0.03a	69.01 ± 6.50a	7.22 ± 0.59a	33.79 ± 6.59c	62.49 ± 4.01b

## Supplementary Materials

Supplementary material is available online at http://www.mdpi.com/1422-0067/19/12/3934/s1.

## Abbreviations

ETo/ABS	quantum yield for electron transport
ETo/TRo	probability that an electron moves further than primary acceptor of PSII
Fv/Fm	the maximal quantum yield of PSII
g_s_	stomatal conductance
MDA	malondialdehyde
NPQ	non-photochemical quenching
Pn	photosynthetic rate
PSI	Photosystem I
PSII	Photosystem II
Q_A_	primary quinone
ROS	reactive oxygen species
Tr	transpiration rate
1-qP	excitation pressure of PSII
ΔMR/MR_0_	the maximal photochemical capacity of PSI
ΦPSII	actual photochemical efficiency of PSII
