# Supplementary material for: Root Abscisic Acid Contributes to Defending Photoinibition in Jerusalem Artichoke (Helianthus tuberosus L.) under Salt Stress
Source: Int J Mol Sci. 2018 Dec 7;19(12):3934. doi: 10.3390/ijms19123934 (PMC6321411; doi:10.3390/ijms19123934)
Supplement: Supplementary file 1 [file ijms-19-03934-s001.pdf]

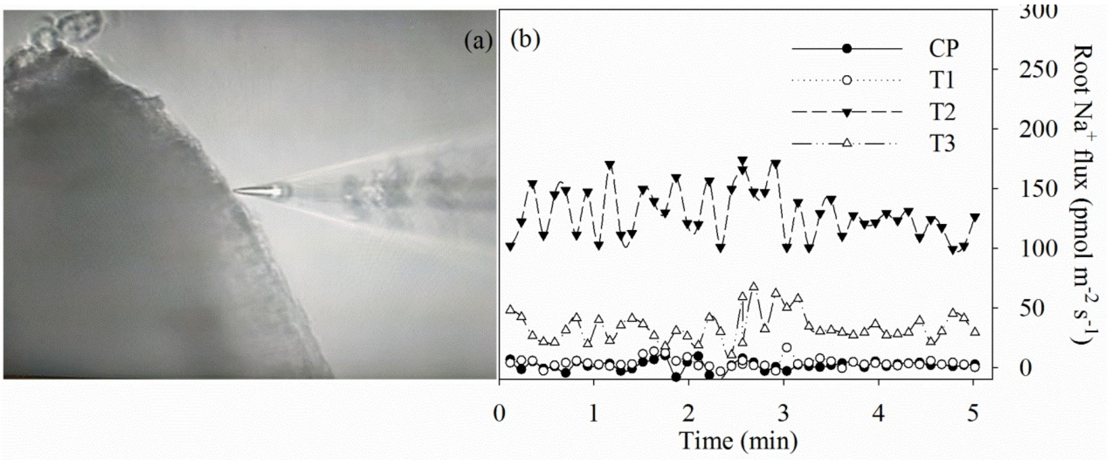

Figure S1. The non-invasive ion-selective electrode closed to the root (a) and net Na^+ fluxes at the point of 500 μm from root apex (b). Na^+ fluxes were measured for 5 min, and each point is the mean of five replicate plants.
